# Psychomotor Retardation in Elderly Untreated Depressed Patients

**DOI:** 10.3389/fpsyt.2014.00196

**Published:** 2015-01-26

**Authors:** Lieve Lia Beheydt, Didier Schrijvers, Lise Docx, Filip Bouckaert, Wouter Hulstijn, Bernard Sabbe

**Affiliations:** ^1^Collaborative Antwerp Psychiatric Research Institute (CAPRI), Antwerp University, Antwerp, Belgium; ^2^University Psychiatric Center KU Leuven, Kortenberg, Belgium

**Keywords:** major depression, elderly, psychomotor retardation, cognition, copying tasks, neuropsychological assessment, medication free

## Abstract

**Background:** Psychomotor retardation (PR) is one of the core features in depression according to DSM V (1), but also aging in itself causes cognitive and psychomotor slowing. This is the first study investigating PR in relation to cognitive functioning and to the concomitant effect of depression and aging in a geriatric population ruling out contending effects of psychotropic medication.

**Methods:** A group of 28 non-demented depressed elderly is compared to a matched control group of 20 healthy elderly. All participants underwent a test battery containing clinical depression measures, cognitive measures of processing speed, executive function and memory, clinical ratings of PR, and objective computerized fine motor skill-tests. Statistical analysis consisted of a General Linear Method multivariate analysis of variance to compare the clinical, cognitive, and psychomotor outcomes of the two groups.

**Results:** Patients performed worse on all clinical, cognitive, and PR measures. Both groups showed an effect of cognitive load on fine motor function but the influence was significantly larger for patients than for healthy elderly except for the initiation time.

**Limitations:** Due to the restrictive inclusion criteria, only a relatively limited sample size could be obtained.

**Conclusion:** With a medication free sample, an additive effect of depression and aging on cognition and PR in geriatric patients was found. As this effect was independent of demand of effort (by varying the cognitive load), it was apparently not a motivational slowing effect of depression.

## Introduction

Apart from a depressed mood and lack of interest, psychomotor symptoms are core features of a major depressive episode (2). Recently, a three factor model of depression was found, representing negative effect, anhedonia, and psychomotor change (3). This psychomotor change symptom cluster has an important clinical, diagnostic, pathophysiological, and therapeutic significance in the clinical and scientific approach of Major Depressive Disorder (MDD) (4–8). Psychomotor retardation (PR) has repeatedly been denoted as an important marker of the melancholia subtype of depression (5, 9–11), and as a predictor for treatment response to several types of antidepressant treatment (5). Since psychomotor functioning is the only factor of depression that does not correlate with severity of depression and since it is not predictive for clinical outcome, it is thought to be a dimension defining a separate type (3), though not exclusively the melancholic subtype of depression. PR has been found to be present in other subtypes of depression too (11–19). However, it is not only the presence of PR that is important, the type of slowing and the cognitive share in the PR are thought to be differentiating between subtypes of depression too (20). Hence the importance of investigating psychomotor functioning in depression in relation to cognitive functioning.

Psychomotor retardation appears to be a particularly predominant symptom of late life depression, an organic subtype of geriatric depression with vascular damage of frontal–subcortical circuits and a depressive–executive dysfunction syndrome (21, 22), but also of other atypical depression presentations such as subsyndromal depression (23). As aging itself already causes a substantial psychomotor slowing in healthy elderly (24–26), elderly depressed patients could be expected to show an even more pronounced form of PR. Pier and colleagues (25) hypothesized an additive effect of aging and depression on the psychomotor performance, be it on the basis of a sample of 11 medicated patients. Bonin-Guillaume et al. (27) too found an additive PR effect in 16 patients. The retardation showed to be an addition of two different types of slowing. There was a general slowing in aging, affecting all stages of information processing, and a more specific slowing in depression, affecting the decisional stage and the neuromotor stage, but not the sensory-motor stage. It should be noted, however, that they did only investigate the reaction time and not the motor time as a measure of psychomotor speed (27). The included patients in both studies were all using psychotropic medication, i.e., antidepressants (selective serotonin re-uptake inhibitors and tricyclic antidepressants) as well as anxiolytics and confounding medication effects were observed (25, 27). Admittedly, polypharmacy is very common in elder age patients, and since these patients are also more sensitive to all kinds of adverse medication induced side-effects, differentiating between the specific effects of depression, age, and medication is particularly difficult, especially as the medication profiles of the subjects in previous studies may have been extremely divergent. Studies on PR in elderly depression are still scarce and show only partial results, because most of these have only measured PR on the basis of cognitive reaction times, without distinguishing and separating out motor slowing (27–30). The two studies that do investigate motor time include only medicated patients (25, 31). All in all, differentiated research of psychomotor symptoms in geriatric depression is still very limited and only exists in medicated clinical cohorts, so that evidence is still missing for the value of these types of symptoms as a diagnostic tool for this subgroup of depressed patients.

Psychomotor retardation not only involves motor processes, but also cognitive processes. Indeed, the term PR “not only encompasses the output of muscle contractions, but also the wider involvement of perceptual processes and cognitive-control mechanisms” (5) (p. 14). Indeed, several cognitive sub-processes contribute to the psychomotor processing. Studies on neuropsychological functioning in late life depression generally mention processing speed and executive function as the main cognitive impairments in MDD in the elderly (32, 33). Yet, PR and executive functioning are not correlated, indicating that cognitive retardation is not the sole explanation of PR (34). It has been suggested that retardation in executive function is merely the consequence of reduced processing speed (35–37). However, Sexton et al. (38) found that executive deficits could not be fully explained by general impairments in processing speed. Controlling for processing speed, Dybedal et al. (32) still found impaired executive function in elderly depressed compared to healthy controls. Considering that both processing speed and executive functioning are the cognitive hallmarks of depression, they will be treated separately here in relation to psychomotor measures. Since executive function and PR are not correlated, it would be interesting to figure out whether depression severity without interfering medication effects, has a specific impact on cognitive and psychomotor functioning, respectively.

The current study aims to measure cognitive and psychomotor functioning in a sample of unmedicated depressed elderly, applying objective psychomotor, and cognitive assessment methods. In accordance with previous studies (25, 28), it is hypothesized that unmedicated elderly depressed patients will perform worse both on the cognitive and psychomotor tasks. Different cognitive and psychomotor measures will be applied to shed a light on different cognitive factors that may influence PR, most importantly processing speed, but also inhibition and interference resistance, cognitive flexibility, and memory. With the objective measures of PR, the cognitive reaction time, i.e., the initiation time of a movement and the reinspection time, the time needed to verify the stimuli, will be separated from the motor time, i.e., the real movement time. Finally, the effect of cognitive load in PR will be tested by experimentally varying the complexity of the stimuli of the copying task to investigate the interaction of cognition and motor functioning in PR.

## Materials and Methods

### Study population

Twenty-eight non-demented (Mini Mental State Examination Score > 24) elderly (age >60) in- and out-patients with unipolar single episode or recurrent MDD, meeting DSM-IVTR criteria (2), were compared to 20 healthy controls, matched for age, gender, education, and vascular risks (diabetes, hypertension, smoking, obesity, and hyperlipidemia). Patients with a MMSE score under 24, the consensus cut-off score for probable dementia (39–41), were excluded. Depression was identified using the DSM-IVTR criteria and the severity of depression was assessed by means of the Geriatric Depression Scale (GDS). A minimum score of 11 on the GDS was required for inclusion of patients. Patients taking medication with important psychotropic impact such as psychopharmacological treatments, but also antihistaminics and anticholinergics for instance, were excluded. For every type of disallowed or concomitant medication, the drug free period before testing was specified. For most antidepressants, a wash-out period of 1 week prior to baseline was applied, with the exception of fluoxetine (5 weeks), fluvoxamine (2 weeks), and monoamine oxidase inhibitors (2 weeks). Any anxiolytics (including benzodiazepines) and hypnotics (except Zolpidem, Zopiclone, or Zaleplon) were disallowed within the last week prior to testing. Patients and controls suffering from any medical condition [e.g., Parkinson’s disease, dementia, psychotic disorders, mental retardation, substance- or alcohol abuse, organic mental disorders due to a general medical condition as defined in the DSM-IV-TR (2)] that might affect fine motor or cognitive processes were excluded, as were patients with personality disorders that might compromise the study. All participants were native Dutch speakers and had given their informed consent after the study was fully explained to them. The study was carried out consistent with the latest version of the Helsinki Declaration (42) and was approved by the medical ethics committee of the participating hospitals.

### Assessments and tasks

All participants performed an extensive cognitive and psychomotor test battery (see below). All testing, for patients and for healthy controls, took place in the afternoon.

#### Clinical assessment

Clinical depression severity was assessed using the GDS (30 items) (43) whereas the State and Trait Anxiety Inventory (STAI 1 and STAI2) (44) informed about the degree of subjective anxiety symptoms. Both tests were also applied to the controls. The 15-item Salpêtrière Retardation Rating Scale (SRRS) (45) was administered to assess the subjective, rated level of PR.

#### Psychomotor tasks

**Figure 1:**
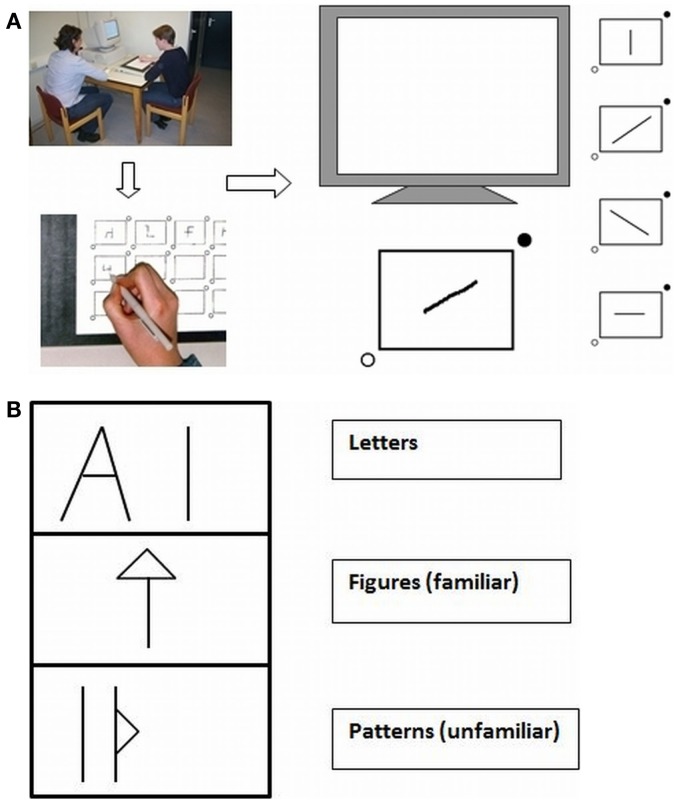
**(A,B)** Set up **(A)** of the line and complex figure copying task **(B)** with pressure-sensitive digitizer.

For the objective psychomotor assessment (46, 47), participants carried out drawing tasks. Subjects were asked to copy figures from a computer screen with the use of a special pressure-sensitive pen and a digitizer (48). A full description of the set up as shown in Figures 1A,B can be found in Pier et al. (25).

##### Line and figure copying task

In the Line Copying Task (LCT; Figure 1A), patients had to draw a line in one of four directions (horizontal, vertical, or one of the two diagonals) as quickly as possible. In the Figure Copying Task (FCT; Figure 1B), they had to copy figures consisting of four line segments with varying complexity, some were well-known letters, other were familiar figures and the third kind were less familiar patterns. The stimulus (line or figure) appeared on the screen when the participant placed the pen tip in the start circle at the bottom left of the box in which the stimulus had to be copied. As soon as participants started drawing, the figure disappeared from the screen. However, there was the possibility (which was not encouraged) to reinspect the figure by retouching the start circle. To end a trial, participants had to place the pen in the stop circle at the upper right corner of the box. Initiation time, the time between the presentation of the stimulus and the start of the first drawing movement, was measured. Also the motor time, the time from the start of the first drawing movement to the end of the last drawing movement, was calculated. In the second task, the reinspection time, the time from retouching the spot to resuming starting the drawing was also determined. Time to reinspect was not included in the motor time.

##### Symbol-digit substitution test

The same recording techniques were used as with the copying tasks (49–51). This made it possible to differentiate between a cognitive and a psychomotor component apart from the general measure of information processing speed. The subjects had to substitute symbols by digits during a period of 90 s, using a key consisting of nine symbol–digit pairs. The following variables were analyzed: raw scores, i.e., the number of correct answers, matching time, representing mean pen-up time, and pause time between two successive digits (comparable to the initiation time in the copying tasks), and writing time, representing the time needed to write a digit (comparable to motor time).

#### Cognitive tasks

##### Wisconsin card sorting task

In the Wisconsin card sorting task (WCST) (52), which is primarily intended to measure cognitive flexibility, an executive function, four key cards were presented with geometric figures that vary according to three perceptual dimensions (color, form, and number). The subjects had to discover the correct sorting principle by trial and error. After each choice they got a feedback (right or wrong). Once the participant made a correct choice, this sorting principle had to be maintained across changing stimulus conditions while ignoring the other – now irrelevant – stimulus dimensions. After 10 consecutive correct matches, the classification principle changed without warning. As the WCST is not timed, sorting continued until all cards were sorted or a maximum of six correct sorting criteria had been reached. Index of the participant’s performance was the number of categories completed (53–56). However, since some patients did not even complete one category, executive functions such as switching could not be measured.

##### Stroop color-word test

The Stroop color-word test (57, 58) is a cognitive test that requires participants to firstly read the names of colors printed in black ink (trial 1), then name printed colors (trial 2) as quickly as possible without making errors and then naming the color of a word in which it is printed (trial 3). The test measures the individual’s ability to suppress task-irrelevant responses (i.e., the tendency to read the color name rather than name the color) and ability to maintain attention and concentration (59). The Stroop interference score was calculated as the time taken to name colors in trial 3 minus the time taken to name color names in trial 2. A higher Stroop interference score thus refers to the degree of interference caused by suppressing the habit of reading words in order to name colors; a higher score reflects poorer performance (59).

##### 15-Words tests

In the 15-words task, a verbal memory task (60), subjects were presented 5 times a list of 15 words, which they had to reproduce. After an interval of 20 min, the experimenter asked to reproduce the memorized words once more. Afterward they had to recognize in a list of 30 words, which were the words they had studied. Only the sum of correct recalls has been recorded (Verbal Memory Total). The delayed recall was scored as Verbal Memory Recall. For the Verbal Memory Recognition too, only correct recognitions were scored.

### Statistical analysis

Statistical analysis of the data was performed using SPSS 17.00 and consisted of a General Linear Method (GLM) multivariate analysis of variance to compare the psychomotor and cognitive outcomes of the two groups. To measure the effect of cognitive load in the figure copying tasks, a GLM Repeated Measures Analysis of Variance with Group (MDD, Controls) as between-subjects factor and Complexity (letters, figures, and patterns) as within-subjects factor was performed. In addition, bivariate Pearson correlations were computed between severity of depression and the other clinical, cognitive, and psychomotor measures. Significance level was set at *p* < 0.05.

## Results

### Demographic and clinical variables

As can be seen in Table 1, there were no significant differences between groups on demographical variables. Patients were significantly more depressed, more anxious (as well state as trait anxiety), and showed more psychomotor retardation (SSRS) and cognitive impairment (MMSE). Severity of depression correlated with none of the cognitive and psychomotor measures, only with clinical measures of state anxiety (r GDS-STAI I = 0.524, *p* = 0.006) and slightly with the clinical rating of retardation (r GDS-SRRS = 0.418, *p* = 0.047).

**Table 1:** **Demographic and clinical variables of patients and controls**.

	Patients (*N* = 28)	Controls (*N* = 20)	*F*	*p*	Cohen’s *d*
Age	74.71 (7.56)	71.95 (5.14)	2.01	0.163	
Male/female	4/24	5/15	*X*^2^ = 0.879	0.348	
MMSE	25.52 (3.80)	28.30 (1.38)	9.73	0.003	0.97
GDS	17.58 (4.46)	4.15 (2.50)	145.83	<0.001	3.71
STAI 1	51.93 (11.38)	34.50 (7.83)	34.98	<0.001	1.82
STAI 2	51.00 (10.25)	34.45 (7.65)	36.81	<0.001	1.83
SRRS	16.44 (8.74)	2.30 (1.92)	50.16	<0.001	2.23

*Standard deviations are shown in parentheses*.

### Cognitive and psychomotor performance

Patients performed significantly worse than controls on all cognitive measures. For an overview, see Table 2. The largest effects are found for the number of correct filled in items on the symbol-digit substitution test (SDST) (Cohen’s *d* = 1.37) (61), the matching time of SDST (Cohen’s *d* = 0.94), the Wisconsin number of categories completed (Cohen’s *d* = 1.40), and the total recall of the verbal memory test (Cohen’s *d* = 0.96). The measures of the perseverative errors and non-perseverative errors in the Wisconsin task had to be left out because they proved meaningless, as patients could not even complete one category. The impaired learning capacity is confirmed by the verbal memory scores. As can be seen in the table, the Stroop tasks too almost reached significance on the 0.01 level. In general, however, the significance was lowered by the difference in variance between patients and healthy controls, with a larger variance in the patient scores, except for the WCST. The latter exception can presumably be explained by a floor effect, as patients did not even manage to learn one category. The difference in SDST total correct, the measure of processing speed, reveals that a general retardation of processing speed is a central feature of elderly depression. Still on the SDST, both the matching and the writing time were significantly higher in patients, indicating cognitive as well as psychomotor slowing on this task.

**Table 2:** **Mean performance levels of patients and controls on cognitive and psychomotor measures**.

	Patient	Control	*F*	*P*	Cohen’s *d*
**Neuropsychological tests**
SDST number correct	43.63 (9.38)	27.52 (13.46)	19.41	<0.001	1.37
SDST_matching time	3.42 (2.90)	1.47 (0.45)	8.44	0.006	0.94
SDST_writing time	1.17 (1.08)	0.66 (0.13)	4.23	0.047	0.67
Stroop card 1	63.43 (24.10)	47.32 (11.21)	7.19	0.011	0.83
Stroop interference	111.43 (110.54)	46.11 (21.42)	37.23	0.016	0.80
WCST *N* categories completed	0.65 (0.83)	2.00 (1.12)	19.16	<0.001	1.40
Verbal memory total	26.71 (11.91)	36.32 (7.77)	9.55	0.003	0.96
Verbal memory recall	4.59 (3.24)	6.63 (3.06)	4.63	0.037	0.53
Verbal memory recognition	22.72 (4.21)	25.72 (2.61)	7.15	0.011	0.86
**Psychomotor tasks**
LCT_initiation time (s)	1.46 (1.00)	0.97 (0.17)	4.49	0.040	0.65
LCT_movement time (s)	0.73 (0.38)	0.47 (0.17)	7.78	0.008	0.86
FCT_initiation time (s)	2.98 (1.03)	2.60 (0.85)	1.67	0.203	0.39
FCT_reinspection time (s)	0.41 (0.66)	0.10 (0.19)	3.99	0.053	0.60
FCT_movement time (s)	3.94 (2.36)	2.38 (1.15)	7.03	0.011	0.79

*Standard deviations are shown in parentheses*.

As for performance on the copying tasks, patients’ initiation time was found to be impaired on the LCT, but not on the FCT, whereas movement time was significantly higher in patients than in controls on both the LCT and the FCT. Analysis reveals a more significant difference between the healthy and the depressive elderly on the movement time compared to the initiation time. Finally, patients reinspected significantly longer than controls on the FCT.

**Figure 2:**
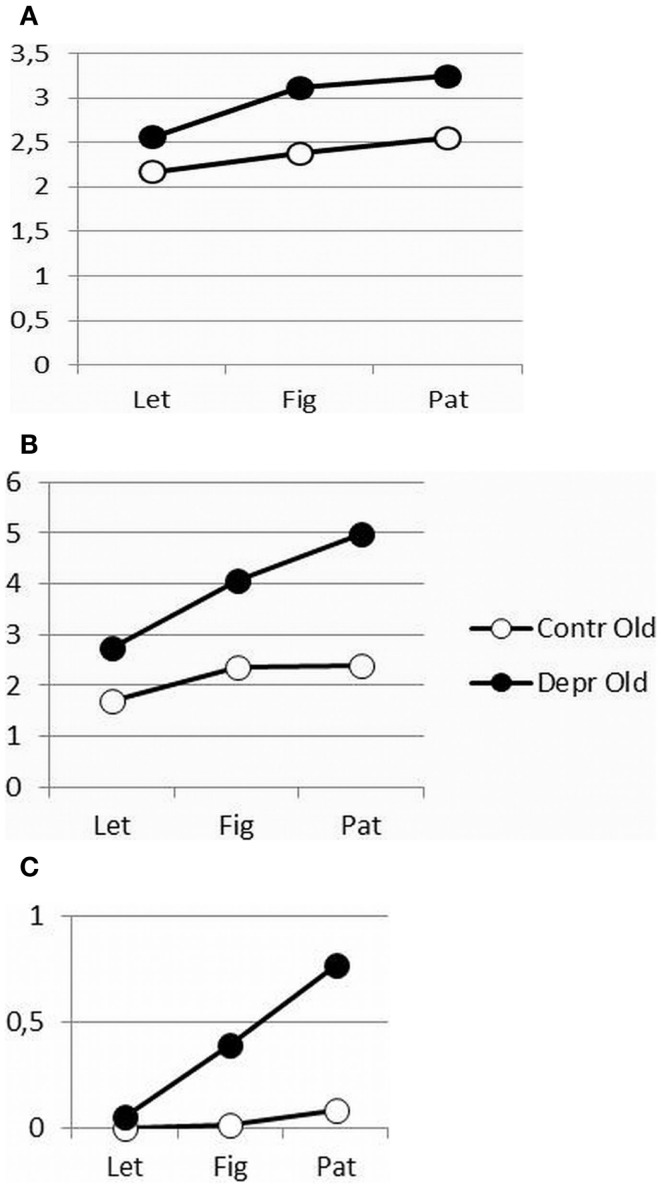
**(A–C)** Differences in initiation time **(A)**, movement time **(B)**, and reinspection time **(C)** as a function of complexity between depressive patients and healthy controls.

As shown in Figure 2, increasing figure complexity in the FCT for increased cognitive load, resulted in a significantly increased initiation time (*F* = 10.38, *p* = 0.0002) and execution time (*F* = 10.721, *p* = 0.0002) for both patients and controls and in a significantly longer reinspection time (*F* = 3.89, *p* = 0.029) in the patient group. However, the increased cognitive load affected patients’ psychomotor performance more than that of controls, except for the initiation time (IT, Figure 2A: *F* = 1.27, *p* = 0.267, ns; MT, Figure 2B: *F* = 10.721, *p* = 0.002; and Reinspection, Figure 2C: *F* = 4.98, *p* = 0.031). Both patients and healthy controls initiated the drawing movements immediately, but the patients faltered while drawing and recurred more often to the stimuli.

## Discussion

In this study, we investigated psychomotor and cognitive performance as an effect of depression in an elderly medication free depressed sample, with both objective motor and cognitive measures. To find out the impact of a cognitive factor in PR, we experimentally varied the amount of cognitive load in psychomotor functioning. Because Tarbuck and Paykel (28), on the basis of an unmedicated sample, assumed that retardation due to age is associated with timed tasks only and that PR due to depression is associated with the complexity of the task, we chose to use a non-timed psychomotor task to see whether the difference still showed. The geriatric depressed patients (as a group) were found to be significantly slower on almost all psychomotor measures, as reflected in high SRRS scores as well as in inferior outcomes on most of the copying tasks, compared to the outcomes recorded for the matched healthy controls. In general, this is in line with previous studies in depressed samples that applied the same assessment methods, in elderly (25) and in younger patients (14, 62–64). However, the sample in this medication free population shows peculiarities of slowing that, moreover, provide valuable insights into the very specific interaction of cognitive and psychomotor slowing in the convergence of depression and aging.

When varying the complexity of figures to copy and thus varying the cognitive load, it is strikingly the motor time that shows the most significant interaction effects of group (depressive elderly versus healthy elderly) and complexity; the reinspection time is less significant, the initiation time not at all. Patients start copying immediately, irrespective of the complexity of the task. Nevertheless, in cognitive more difficult motor tasks, the movements of the depressed elderly become slower or more hesitating, with some more reinspection. Apparently, various cognitive and motor processes are involved in figure copying. Initiation times are assumed to mainly reflect the cognitive processes and encompass the attention for and the perception of the stimulus figure, as well as the storage of the representation in working memory, but also the programing and planning of the first drawing movement and the activation of motor programs that initiate the muscle to start drawing (14).

Scrutinizing the differential effect of increased task difficulty on movement time and initiation time leads to an adaptation of the notion of initiation time. Traditionally, “initiation time” has been defined as a “cognitive” time, different from “motor” time, the time of execution of the movement (14, 25, 64). The fact that more complex tasks lead to longer motor times but not to longer initiation times reveals a cognitive aspect in the motor time. The initiation time, in turn, should be perceived as a simple reaction time, a measure of general processing speed and cognitive reserve. This measure of processing speed in the performed tasks was merely measuring the time of “decision to start,” which is not different for simple and complex tasks. “Decisions are made by accumulating noisy stimulus information until sufficient information for a response [for a response criterion] is obtained” (65). Admittedly, the initiation time of patients was longer compared to controls in copying simple lines. This could, however, be linked to changes in white matter integrity of the motor system (66). In a study of Walther et al. (66), patients with MDD differed from healthy controls in a loss of frontal integrity, which was linearly related to a lower activity level. An alternative explanation could be that there was already a ceiling effect of slowing of initiation time in simple tasks in patients. Slower subjects have already more influence of prefrontal executive control in simple tasks for successful performance (67). Evidence has been found indeed for different associations between structures and behavior in depressive patients and healthy controls (66). Bracht et al. found altered cortico-cortical white matter motor pathways, and concluded that these may contribute to movement initiation in MDD (68). A more refined gradation of cognitive reserve impairment can still be made by involving the motor time assessed in the copying task. The execution of a movement while planning and preparing the next is a dual task recruiting more brain regions in parallel (69). The impaired efficiency of interaction between the dorsolateral prefrontal cortex and the pre-supplementary motor area by altered white matter organization of the pathway (68) needs more prefrontal executive higher order compensation (69). We presume that this hierarchical plasticity of the brain principle with higher order integration for output with lower order deficits is also responsible for disbalanced motor control with more activation of (higher order) right orbitofrontal cortex and less activation of the (lower order) left supplemental motor area in higher activity level (70).

The predominantly dopaminergic dysregulation of cognition and motor functioning by striatal dopamine transporters (71, 72) has motivational correlates too (73), manifested in decisional anhedonia (74). Lowered mesolimbic dopamine projections in the nucleus accumbens (74) and overstimulation of nucleus accumbens adenosine receptors (75) change the GABAergic signals that relay through the ventral tegmentum, associated with motor control, and the substantia nigra, associated with reward cognition, resulting in changed effort-based decision making with decreased perceived net-value under increasing response costs (74). These response costs can be increased by task complexity or higher activity level. Limitations of these findings are the important age and sex influences in dopaminergic neuromodulating influences (72), which urge for further investigation.

Clearly, figure copying is different from the separate cognitive measures in standard cognitive testing. Even the SDST tends to reflect higher order cognitive, memory related functions more than it does psychomotor speed (14, 76). The bigger higher order executive cognitive load of searching for a number in the legend code, memorizing the found digit and subsequently performing the initiation and planning of writing the digit in the SDST and the relative easiness of writing a well-known automatized digit compared to an unknown pattern, may also explain the difference in effect size of the matching time of SDST (Cohen’s *d* = 0.94) and the initiation time of the figure copying (Cohen’s *d* LCT initiation = 0.65; Cohen’s *d* FCT initiation = 0.39). Furthermore, patients performed worse than controls on all cognitive measures in the standard cognitive tasks. It must be remembered, however, that above all, the more cognitive executive aspects show cumulative effects of aging and depression, except in the WCST. The lack of interaction effect in the WCST is clearly a result of the missing measure of executive function due to the patients’ inability to learn even one category. Measuring adaptation and perseveration thus became impossible.

All in all, the difference in slowing as a result of increasing cognitive load may be explained as an effect of cognitive aspects in psychomotor functioning. Presumably, the cognitive component of PR is different in nature and involves more motor circuitry involvement than that measured by the standard cognitive tasks.

**Figure 3:**
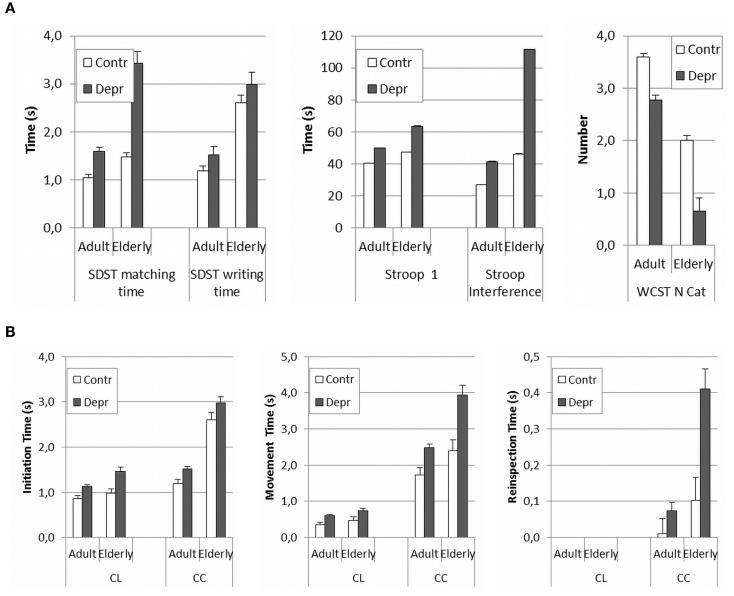
**(A,B)** Comparison of psychomotor and cognitive measures between healthy and depressed elderly against the background of previous research with the same tasks in adults. Because of limited competence of the population, with the elderly the copying task consisted of just four lines, whereas with the adults a task with eight lines was used. To make the results comparable, recalculations were made for the adult scores based on the mean time for four lines. Separate times for each line were available.

The present results suggest that PR observed in the patient group was caused by both a cognitive and a motor factor, as, respectively, most matching times and writing times were higher in patients. In order to further scrutinize the possible cognitive effect, we compared the current results *post hoc* to the ones obtained in a similar study in an adult population of depressed medicated patients and in healthy controls (18–60 years). This way, we could also gain some insight into possible interaction effects of age and depression and we could determine whether there was a link with cognitive functioning. In Figure 3, we have presented the results of this *post hoc* comparison. Since adult medicated patients appear to be even less retarded than elderly depressive unmedicated patients, these results only corroborate the hypothesis of an aging effect in depression. The overall comparison in Figure 3 reveals a clear effect of depression in all ages, both, for the cognitive measures (Figure 3A: F SDST matching time = 36.40, *p* < 0.001; F SDST writing time = 22.36, *p* < 0.001; F Stroop card 1 = 25.58, *p* < 0.001; F Stroop interference = 31.24, *p* < 0.001; and F WCST N categories completed = 10.54, *p* = 0.001) and for the psychomotor measures (Figure 3A: F LCT initiation time = 24.29, *p* < 0.001; F LCT movement time = 13.83, *p* < 0.001; F FCT initiation time = 8.54, *p* = 0.004; F FCT reinspection time = 14.71, *p* < 0.001; and F FCT movement time = 25.35, *p* < 0.001). An aging effect is equally obvious, also in both, in cognitive measures (Figure 3A: F SDST matching time = 29.96, *p* < 0.001; F writing time = 45.32 *p* < 0.001; F Stroop card 1 = 16.21, *p* < 0.001; F Stroop interference = 39.19, *p* < 0.001; and F WCST N categories completed = 31.21, *p* < 0.001) and in psychomotor measures (Figure 3B: F LCT initiation time = 8.55, *p* = 0.004; F LCT movement time = 3.22, *p* = 0.074; F FCT initiation time = 144.70, *p* < 0.001; FCT reinspection time = 19.37, *p* < 0.001; and FCT movement time = 22.02, *p* < 0.001). A calculation of possible interaction effects of aging and depression in the GLM test indicates that only the matching time and the writing time of the SDST and the Stroop interference show interaction effects (F SDST matching time = 11.80 *p* = 0.001; F SDST writing time = 9.50, *p* = 0.002; F Stroop card 1 = 1.57, *p* = 0.211; F Stroop interference = 12.65, *p* < 0,001; F WCST = 0.63, *p* = 0.429). In the psychomotor measures, only the reinspection time shows a slightly significant interaction effect (F LCT initiation time = 2.10, *p* = 0.149; F LCT movement time = 0.001, *p* = 0.979; F FCT initiation time = 0.04, *p* = 0.837; F FCT reinspection time = 6.35, *p* = 0.12; and F FCT movement time = 3.09, *p* = 0.80). However, this effect was not reflected in the results. The significance was diminished by the much larger variance on the reinspection times of the complex figure copying task in the elder population. Indeed, there is an overall increase of variance in the elderly, especially in psychomotor tasks where motor and cognitive aspects coincide (SDST matching, writing time, and complex figure reinspection). Overlooking the overall results leads to the assumption that the interaction of depression and aging reveals itself in executive functioning and in the interaction of cognitive and psychomotor functioning. The main comparison of the Cohen’s *d* effect sizes in the elderly and adult group shows that the effect of depression is always bigger in elderly. The relatively small difference between the effect sizes of the adults and the elderly however, is explained by the large variance in older groups, which limits the found intergroup effects. Surprisingly, the effect sizes of initiation time of the copying tasks show the reverse direction; it is bigger in adults. Evidently, these results need to be confirmed by direct comparative research.

The present study not only confirms the results of a similar study by Pier et al. (25), it also provides a valuable contribution in its own right, as it overcomes some of the restrictions of the earlier study. Whereas, the study by Pier et al. (25) was a small sample study (*n* = 11) in which patients were taking medication that could have impacted the results, the present study is unique in that it involves only patients that are free of psychotropics. The importance of the latter condition is apparent from the fact that in the Pier et al. study (25) correlations were found between the use of antidepressants and anxiolytics on the one hand, and several psychomotor outcomes on the other. With our larger medication free sample, we succeeded in replicating the results of Pier et al. (25), corroborating their preliminary results concerning the presence of PR in elderly depressed patients, independent of medication status. Apart from that, the present study revealed an interesting difference between medicated and unmedicated patients. In comparison to the control groups (healthy aged, younger depressed), the pattern of interaction between the degree of slowing and the cognitive complexity of the task in the unmedicated elderly sample seemed to be the reverse. In the unmedicated elderly sample, PR was proportionately more visible in more complex tasks (copying more complex figures, less familiar figures) than in copying simple lines. In the medicated sample, on the contrary, the PR was more obvious in comparison with the other groups in the simple copying task than in the more complex tasks (63) (p. 24). This result is in line with the suggestion by Caligiuri et al. (20) that retardation caused by medication is predominantly neuromotor retardation, i.c. abnormal velocity, as opposed to the psychomotor slowing in depression, in which the cognitive factor is more important. Benzodiazepines, opioids, anticholinergics, but also tricyclic antidepressants (77) often elicit modest or more pronounced psychomotor or cognitive impairments (78). These findings support the diagnostic relevance of the quality of slowing in major depression, in aging and in a broad range of psychopathological disorders.

Notwithstanding the relatively small sample size, the reported effects were robust. The very restrictive inclusion criteria determining the sample size were introduced because of the high comorbidity of depression and the considerable use of medication in the elderly and because of the numerous possible cognitive – and psychomotor – side-effects of somatic and degenerative diseases. To avoid such confounding cognitive effects a selection of elderly depressive patients imposed itself. Despite the fact that such a strict selection can hardly be seen as representative for the “natural” population, it afforded a unique opportunity to rule out possible medication and comorbidity effects and to obtain an unbiased view on the differential PR effects of depression in the elderly. A limitation of this study could be that cardiovascular disease, a recognized cause of psychomotor slowing in elderly due to white matter lesions (WML) (79), was only excluded after introducing a questionnaire for the patient and the treating physician. The sample of patients was, however, too small to introduce cardiovascular disease as a covariate. MRI volume measures of WML could have provided a more objective measure of vascular risks causing neuromotor slowing (80), but the primary focus of the present investigation was to reveal the different types of slowing factors. Admittedly, Hickie et al. (79), who combined clinical, neuropsychological, magnetic resonance imaging, and single photon emission computerized tomography, found that the percentage caudate nucleus regional cerebral blood flow was associated with psychomotor slowing and presence of WML. Even if their neuropsychological measures included only reaction times and did not offer an integrated view of cognitive and motor psychomotor slowing (cf. the movement time of complex figures in the present study), they still found 25% of the variance explained by “depression.” Consequently, they concluded that “while psychomotor slowing is determined in part by subcortical changes, other cortical and illness-dependent factors are likely to be relevant” (79). This result confirms the necessity of a detailed neuropsychological analysis of psychomotor functioning in the explorative stage. Such analysis is essential to unravel the complexity of the symptom of slowing and to better understand its physiopathology. Further research with more direct neurobiological measures will have to objectify relative shares of biological and functional aspects in different types of slowing. Since it has become clear that biological aspects like WML account for age-related declines, irrespective of depression (81), an interdisciplinary approach of PR in elderly depressed seems to be in order.

## Conflict of Interest Statement

This research was supported by a financial grant of Lundbeck.
